# Preparation, Characterization and Evaluation of Drug Release Properties of Simvastatin-loaded PLGA Microspheres

**Published:** 2016

**Authors:** Reza Masaeli, Tahereh S. Jafarzadeh Kashi, Rassoul Dinarvand, Mohammadreza Tahriri, Vahid Rakhshan, Mehdi Esfandyari-Manesh

**Affiliations:** a *Dental Biomaterials Department, School of Dentistry, Tehran University of Medical Sciences, Tehran, Iran.*; b *Department of Pharmaceutics, School of Pharmacy, Tehran University of Medical Sciences, Tehran, Iran.*; c *Biomaterials Group, Faculty of Biomedical Engineering, Amirkabir University of Technology, Tehran, Iran. *; d *Department of Anatomy, Dental Branch, Islamic Azad Univesity. *; e *Nanotechnology Research Center, Tehran University of Medical Sciences, Tehran, Iran.*

**Keywords:** Drug-delivery systems, Simvastatin, Poly (D, L-lactic-co-glycolide) (PLGA), Microspheres, Controlled release

## Abstract

Microspheres formulated from poly (D, L-lactic-co-glycolide) (PLGA), a biodegradable polymer, have been extensively evaluated as a drug delivery system. In this study, the preparation, characterization and drug release properties of the PLGA microspheres were evaluated. Simvastatin (SIM)-loaded PLGA microspheres were prepared by oil-in-water emulsion/solvent evaporation method. The microspheres were then frozen to −80 °C, they were freeze dried for 24 h. Characterization of SIM-loaded PLGA microspheres was evaluated by X-ray diffraction analysis, Fourier transform infrared spectroscopy analysis, and scanning electron microscopy (SEM). Drug release potential was evaluated by UV-spectrophotometry. The experimental results revealed that SIM-loaded PLGA microspheres can be successfully obtained through solvent evaporation method with appropriate morphologic characteristics and high encapsulation efficiency.

The drug release pattern from polymeric microspheres in the phosphate buffered saline medium was measured during a 21-day period using UV-spectrophotometry. The correlation coefficient value (r^2^= 0.9878) of the trend lines of the graph showed that the SIM-loaded PLGA microspheres best fit with zero order release pattern. No burst release was observed with polymeric matrix. The drug release characteristic of the microspheres ascertained that the release was about 27% for SIM-loaded microspheres, which occurred within the first 6 days after maintaining the microspheres in phosphate buffer saline. Also, the microspheres successfully presented a slow release and the duration of the release lasted for more than 21 days. It can be concluded that SIM-loaded PLGA microspheres hold great promise for using as a drug-delivery system in biomedical applications, especially in drug delivery systems and tissue engineering.

## Introduction

Solid polymer biodegradable particulates such as micro and sub-micron spheres are of great interest because of their high dissolution rates, which enhance drug bioavailability and have received a significant amount of attention as drug delivery systems (DDS) due to their potential use in locally targeted pharmaceutical delivery (1), medical applications (2), and regeneration of injured tissues (1, 3). Such microspheres must be easy to produce, biocompatible, and biodegradable in order to be available and effective (1, 4, 5). Moreover, microspheres do not only facilitate repair procedures of injured tissues, but they also contain hollow shells to establish a large surface area for adherent cells to attach and proliferate (1, 6). Among the variety of biomaterial candidates for microsphere development, poly(lactic-co-glycolic acid) (PLGA) microspheres have been most extensively evaluated for drug delivery and tissue engineering applications, due to their biodegradation potential and physiological removal (1, 7, 8).

Several methods have been employed to prepare biodegradable microspheres, containing the solvent evaporation method (9), rapid solvent removal by increasing temperature (10), and the solution induced phase separation (1, 11). Among the variety of techniques, solvent evaporation method is the most common method used to produce biodegradable microspheres aimed at drug and protein delivery (1, 12-16). The experimental procedure of fabricating microspheres using this approach is relatively simple; however, many different parameters could affect the morphology and structure of microspheres, which have been extensively reported (1, 7, 9). The size of spheres, drug encapsulation, and formation of hollow shells are often poorly controllable when using emulsion preparation routes (1, 16). 

Simvastatin (SIM) is a hypolipidemic drug employed to control hypercholesterolemia. Additionally, SIM greatly stimulates bone regeneration; hence it might be loaded into microspheres to possibly improve stability of microspheres after formation (1, 17, 18). Biodegradable and porous microspheres are desirable for DDS applications (1, 19). Hence, we developed SIM-loaded PLGA microspheres using the solvent evaporation method with the purpose of using for DDS. In this study, the preparation, characterization and drug release properties of the PLGA microspheres were evaluated. 

## Experimental

*Preparation of SIM-loaded PLGA microsphere*

PLGA (50:50) was purchased from Boehringer Ingelheim (503 H, Ingelheim am Rhein, Germany). Polyvinyl alcohol (PVA) and dichloromethane (DCM) were purchased from Merck Co (Kenilworth, New Jersey, United States). Simvastatin was obtained from Sigma-Aldrich Co (St. Louis, Missouri, United States).

Simvastatin-loaded PLGA microspheres were prepared by oil-in-water (O/W) emulsion/solvent evaporation method. In brief, 5 and 10 mg simvastatin and 500 mg PLGA were dissolved in 3 mL of DCM; this solution was dropped into 300 mL of 0.25% (w/v) PVA solution at room temperature under magnetic stirrer for 45 min until most of the methylene chloride evaporated, leaving solid microspheres. The microsphere suspension was centrifuged at 3000 rpm for 15 min and the microspheres were washed 3 times with distilled water. The microspheres were then frozen to −80 °C, and were freeze dried for 24 h using a freeze dryer.

*Characterization of SIM-loaded PLGA microsphere*

*X-ray diffraction (XRD) analysis*

XRD patterns were obtained at room temperature using a very high-resolution Cu-K_α_ radiation diffraction system (Equinox3000, INEL, Artenay, France) operating at a voltage of 40 kV and current of 30 mA. CPCs were analyzed in the 2θ angle range of 0–80°.

*Fourier transform infrared spectroscopy (FTIR) analysis*

Infrared spectroscopy was carried out to determine the chemical composition of the prepared microspheres using FTIR (Nicolet, USA) operating in the wavenumber range of 400–4000 cm^-1^ at the absorption mode.

*Scanning electron microscopy (SEM)*

The prepared microspheres were coated with a thin layer of gold by sputtering (Emitech K450X, England) and then the microstructure were observed in a scanning electron microscope (SEM; AIS-2100 780, Seron, South Korea) that operated at an acceleration voltage of 20 kV.

*Encapsulation efficiency*

Total drug content in microspheres was determined by dissolving of 20 mg microspheres in 5 mL acetonitrile followed by the addition of 10 mL of methanol to precipitate the polymer. The sample was then centrifuged for 10 min at 21,000×g and the aliquot was taken from the supernatant and analyzed by a spectrophotometer. The encapsulation efficiency was calculated as the actual drug content divided by theoretical drug content multiplied by 100.

*Drug release evaluation*

SIM drug released from the 0.2 g of porous microspheres were determined in phosphate buffer saline (PBS, 0.15 M, pH 7.4) at 37 °C. Released SIM was determined using a UV-spectrophotometer at a wavelength of 238 nm. A standard calibration curve of known amounts of SIM was used to quantify the amounts of loaded and released SIM.

## Results and Discussion

*XRD analysis*

**Figure 1:**
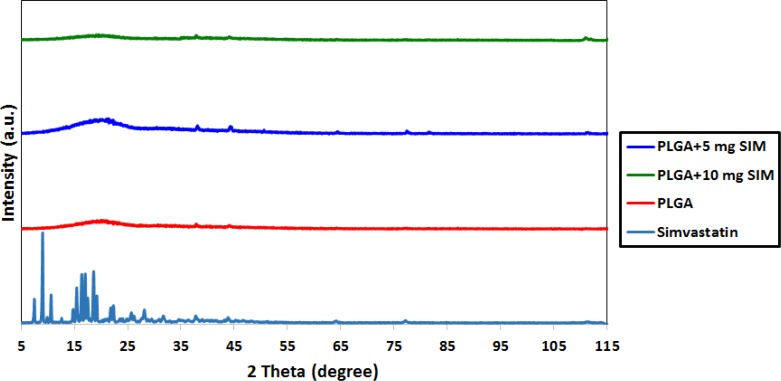
XRD patterns of SIM, PLGA and SIM-loaded PLGA

The XRD patterns of SIM, PLGA, and SIM-loaded PLGA microspheres are shown in Figure 1. Several distinct peaks in the XRD of SIM at diffraction angles of 2 θ; 11°, 13°, 15.5°, 16.5°, 17.5°, 19°, 22.5°, 26°, 28.5° and 32° indicated that SIM was present in a crystalline form. However, these peaks were not observed in the XRD pattern of SIM-loaded microspheres, indicating that SIM would be either molecularly dispersed in the polymer or distributed in an amorphous form.

*FTIR analysis*

**Figure 2:**
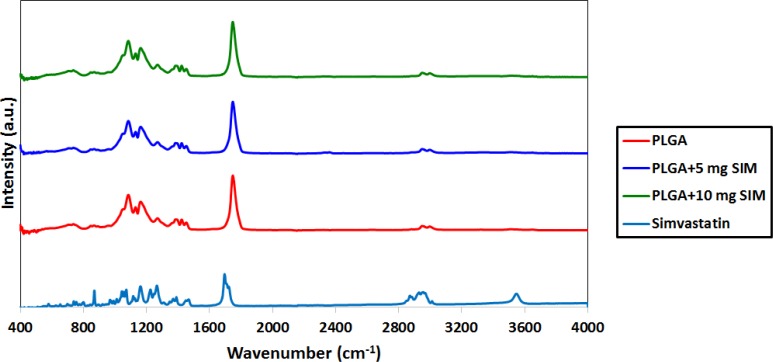
FTIR spectra of SIM, PLGA and SIM-loaded PLGA

Figure 2 shows the FTIR spectra of PLGA, SIM, and the SIM-loaded PLGA microspheres. The PLGA spectrum displays the bands of the OH stretching vibrations (3200–3500 cm^−1^), the -CH, -CH_2_, -CH_3_ stretching vibrations (2850–3000 cm^−1^), the carbonyl C=O stretching vibrations (1700–1800 cm^−1^), and the C–O stretching vibrations (1050–1250 cm^−1^). The FTIR spectrum of SIM displayed prominent absorption at 3553 cm^−1^ (free O–H stretching vibration), 3015, 2964, 2882 cm^−1^ (C–H stretching vibrations), and 1722 cm^−1^ (stretching vibration of ester and lactone carbonyl functional group). These bands were clearly distinct in the SIM-loaded PLGA microspheres; also there were no major shifting, confirming successful SIM incorporation. 

*SEM observations*

**Figure 3:**
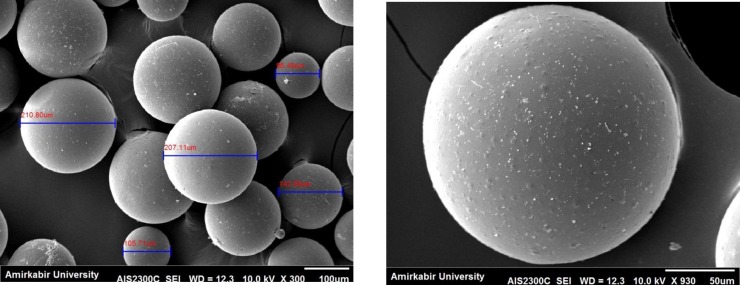
SEM micrographs of SIM-loaded PLGA

The SEM micrographs of the SIM-loaded microsphere are presented in Figure 3. It is evident that the morphology of the microspheres is homogeneous and majority of the spheres are ranging between 105 and 210 µm.

*Drug Loading Efficiency*

Drug loading efficiency of SIM loaded-PLGA microspheres prepared in this study was shown to be approximately 85%.

*Drug release evaluation*

Drug release from biodegradable polymeric particles occurs through a combination of several mechanisms. It generally occurs through desorption of surface-bound drug, diffusion of the drug through the polymer matrix, and erosion of the polymer particles.

**Figure 4:**
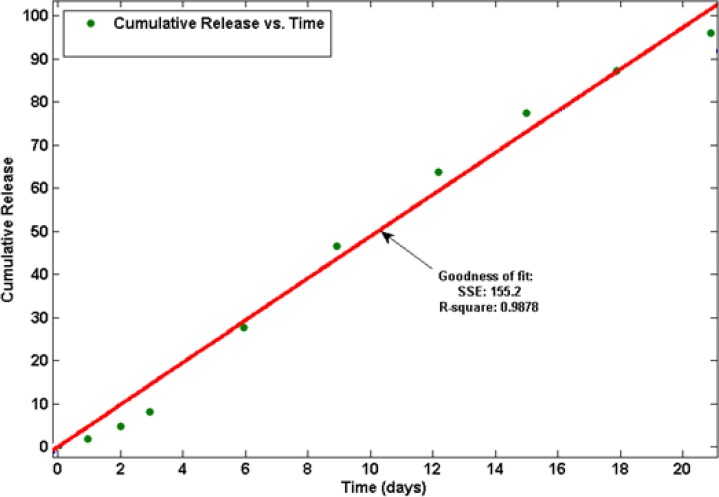
Drug release profile of SIM from PLGA microspheres, (goodness of curve fit is represented as the parameter R-square).

In this study, it is observed linear prolonging drug release rates for more than 21 days without initial burst release (see Figure 4). 

Mean ± standard deviation; n = 3

**Figure 5:**
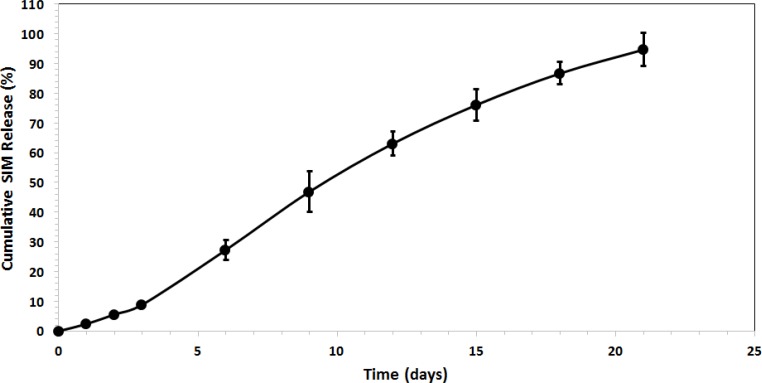
Drug release profile of SIM from PLGA microspheres.

 We found that the release was about 27 % for SIM-loaded microspheres, which occurred within the first 6 days after maintaining the microspheres in PBS (see Figure 5). No initial burst release suggests low drug density at the surface of the microspheres. This could happen due to the similar solubility of the polymer and the SIM in the solvent which means drug was distributed among the microspheres quite homogenously. The rate of drug release gradually increased after about 3 days (about 9 % of the loaded SIM at the beginning). The later constant release is mainly due to drug diffusion and matrix erosion mechanisms of biodegradable PLGA polymer (20).

Mean ± standard deviation; n = 3

The kinetics of drug release from microspheres is closer to the Zero order model. The correlation coefficient (r^2^) was 0.9878 and the curve was fitted to Zero order kinetic (21). This release was due to diffusion and degradation of the microspheres. The initial from solvent emulsification is lower because of the larger size of the microspheres, which means lower surface area and a lower initial.

## Conclusions

SIM-loaded PLGA microspheres were easily and stably prepared employing the solvent evaporation method. XRD analysis demonstrated that SIM would be either molecularly dispersed in the polymer or distributed in an amorphous form. FTIR analysis confirmed that SIM has been incorporated into PLGA microspheres. SEM observations displayed the morphology of the microspheres was homogeneous and majority of the spheres are ranging between 105 and 210 µm. 

SIM-loaded PLGA microspheres showed the prolonged drug release over 21 days without initial burst release.

The drug release results ascertained that the release was about 27% for SIM-loaded microspheres, which occurred within the first 6 days after maintaining the microspheres in PBS. Therefore, obtained experimental results showed PLGA microspheres may be a suitable carrier for control released the SIM for DDS and Tissue engineering.
